# Digital Diabetes Management Technologies for Type 2 Diabetes: A Systematic Review of Home-Based Care Interventions

**DOI:** 10.7759/cureus.84177

**Published:** 2025-05-15

**Authors:** Bassel Abdul Latif el Ejel, Saba Sattar, Syeda Bisma Fatima, Hadequa Noor Khan, Husnain Ali, Abdullah Iftikhar, Muhammad Asad Sarwer, Minahill Mushtaq

**Affiliations:** 1 General Medicine, Stavropol State Medical University, Stavropol, RUS; 2 Internal Medicine, King Edward Medical University, Lahore, PAK; 3 Medicine and Surgery, Abbasi Shaheed Hospital, Karachi, PAK; 4 Medicine, Karachi Medical and Dental College, Karachi, PAK; 5 Medicine and Surgery, King Edward Medical University, Lahore, PAK; 6 Medicine, King Edward Medical University, Lahore, PAK; 7 Internal Medicine, Services Hospital Lahore, Lahore, PAK

**Keywords:** diabetes, digital technology, mhealth, self-management, telemedicine, type 2 diabetes

## Abstract

Digital diabetes management technologies (DDMTs) have emerged as promising tools for improving glycemic control in patients with type 2 diabetes mellitus (T2DM) receiving home-based care. This systematic review evaluates the effectiveness of various DDMTs, including mobile health applications, continuous glucose monitoring (CGM), telemedicine, smart insulin pens, and artificial intelligence-driven decision support systems, in optimizing blood glucose levels. A comprehensive literature search across PubMed, Embase, Scopus, Web of Science, and the Cochrane Library identified nine high-quality systematic reviews published between 2020 and 2024. These reviews synthesized evidence from randomized controlled trials (RCTs) and observational studies, with sample sizes ranging from small pilot studies to large-scale trials. The findings indicate that DDMTs significantly improve HbA1c levels, fasting blood glucose, and postprandial glucose compared to standard self-care practices. Mobile applications and CGM systems demonstrated notable reductions in HbA1c, while telemedicine interventions enhanced patient adherence and engagement. Personalized coaching and real-time feedback were key factors in intervention success. However, challenges such as digital health literacy, cost barriers, and long-term adherence remain concerns. Some studies highlighted the need for sustained engagement to maintain long-term benefits. While DDMTs offer a viable alternative to traditional diabetes management, future research should focus on standardizing interventions, addressing accessibility issues, and evaluating their cost-effectiveness. This review contributes to the growing evidence supporting DDMTs in T2DM management and underscores the potential of digital health innovations in improving glycemic outcomes and patient self-care in home settings.

## Introduction and background

Diabetes mellitus is a global public health concern, with type 2 diabetes (T2D) accounting for over 90% of all diabetes cases worldwide [1]. The prevalence of T2D continues to rise, driven by factors such as aging populations, sedentary lifestyles, and poor dietary habits [2]. Managing blood glucose levels effectively is critical to reducing diabetes-related complications, including cardiovascular disease, neuropathy, and retinopathy. Traditionally, diabetes management has relied on frequent in-person consultations, medication adherence, and lifestyle modifications. However, the increasing burden on healthcare systems has necessitated the exploration of innovative approaches such as digital diabetes management technologies (DDMTs) to enhance self-care and glycemic control, particularly in home-based settings [3].

DDMTs encompass a range of digital tools, including mobile health (mHealth) applications, continuous glucose monitoring (CGM) systems, telemedicine, and artificial intelligence-driven decision support systems [4]. These technologies aim to provide real-time feedback, facilitate patient engagement, and enhance healthcare provider monitoring, thereby improving adherence to treatment regimens and optimizing blood glucose levels. The integration of DDMTs into home-based diabetes care has been associated with improved glycemic control, reduced hospital visits, and enhanced quality of life for patients [5]. Moreover, recent advances in wearable technology and remote patient monitoring have further expanded the potential for personalized diabetes management.

Despite the promising benefits of DDMTs, there remain challenges regarding their effectiveness, accessibility, and long-term adherence. Some studies have shown that while digital interventions may improve short-term glycemic outcomes, sustained benefits require continuous patient engagement and healthcare provider involvement. Additionally, disparities in digital health literacy, cost-related barriers, and concerns about data security present challenges to widespread adoption. Understanding the effectiveness of DDMTs in real-world settings is essential to inform clinical guidelines and optimize diabetes care strategies [3,6,7].

This systematic review aims to assess the effectiveness of DDMTs for blood glucose control in home-based T2D care. By synthesizing evidence from recent studies, this review will evaluate the impact of various DDMTs on glycemic outcomes, patient adherence, and overall diabetes management. The findings will contribute to the growing body of literature on digital health interventions and provide insights into their role in improving diabetes self-management.

## Review

Materials and methods

This umbrella review adheres to the Preferred Reporting Items for Systematic reviews and Meta-Analyses (PRISMA) 2020 guidelines to ensure a systematic and transparent evaluation of existing evidence [8]. It synthesizes findings from previous systematic reviews and meta-analyses to assess the effectiveness of DDMTs in home-based care for T2D mellitus (T2DM). The study follows a structured approach, detailing the eligibility criteria, search strategy, data extraction, and analysis methodology.

Search Strategy

A comprehensive literature search was conducted across major electronic databases, including PubMed, Embase, Scopus, Web of Science, and the Cochrane Library, to identify systematic reviews and meta-analyses relevant to the topic. The search incorporated keywords and Boolean operators (AND, OR) to refine the results. Key search terms included “Type 2 Diabetes,” “Digital Diabetes Management,” “Mobile Health,” “Telemedicine,” “Remote Monitoring,” “Continuous Glucose Monitoring (CGM),” “Smart Insulin Pens,” “Artificial Intelligence in Diabetes,” “Blood Glucose Control,” and “HbA1c Reduction.” The search was limited to English-language studies published between January 1, 2020, and January 01, 2025, to ensure the inclusion of the most recent advancements in DDMTs. Gray literature, conference abstracts, and non-peer-reviewed sources were excluded.

Eligibility Criteria

The study defined clear eligibility criteria to ensure the selection of high-quality evidence. Included studies were systematic reviews and meta-analyses that evaluated DDMTs for blood glucose control in home-based T2DM patients. The population of interest comprised adults (≥18 years) diagnosed with T2DM receiving diabetes care in a home setting. The interventions assessed included mHealth applications, AI-driven glucose prediction models, CGM, smart insulin pens, and telehealth-based diabetes management. These interventions were compared to traditional diabetes self-management strategies, such as standard glucose monitoring and in-person clinical visits. Primary outcomes included changes in glycated hemoglobin (HbA1c) levels, fasting blood glucose (FBG), postprandial glucose (PPG), incidence of hypoglycemia/hyperglycemia, and patient adherence. Studies focusing on type 1 diabetes (T1D), gestational diabetes, or prediabetes were excluded. Additionally, narrative reviews, scoping reviews, editorials, case reports, and opinion pieces, as well as studies lacking relevant blood glucose-related outcomes or those primarily assessing hospital-based interventions, were not considered.

Data Extraction and Synthesis

Two independent reviewers screened the titles and abstracts of retrieved studies, followed by a full-text evaluation of potentially relevant articles. Discrepancies were resolved through discussion or consultation with a third reviewer. A structured data extraction form was used to collect key study characteristics, including publication details, the number and type of primary studies analyzed, DDMTs assessed, reported outcomes, and the methodological quality of each review. A narrative synthesis approach was employed to summarize key findings, highlighting emerging trends, intervention effectiveness, and patient adherence patterns.

Quality Assessment

The methodological quality of the included systematic reviews was assessed using the AMSTAR 2 (A MeaSurement Tool to Assess systematic Reviews) checklist.

Results

Study Selection Process

**Figure 1:**
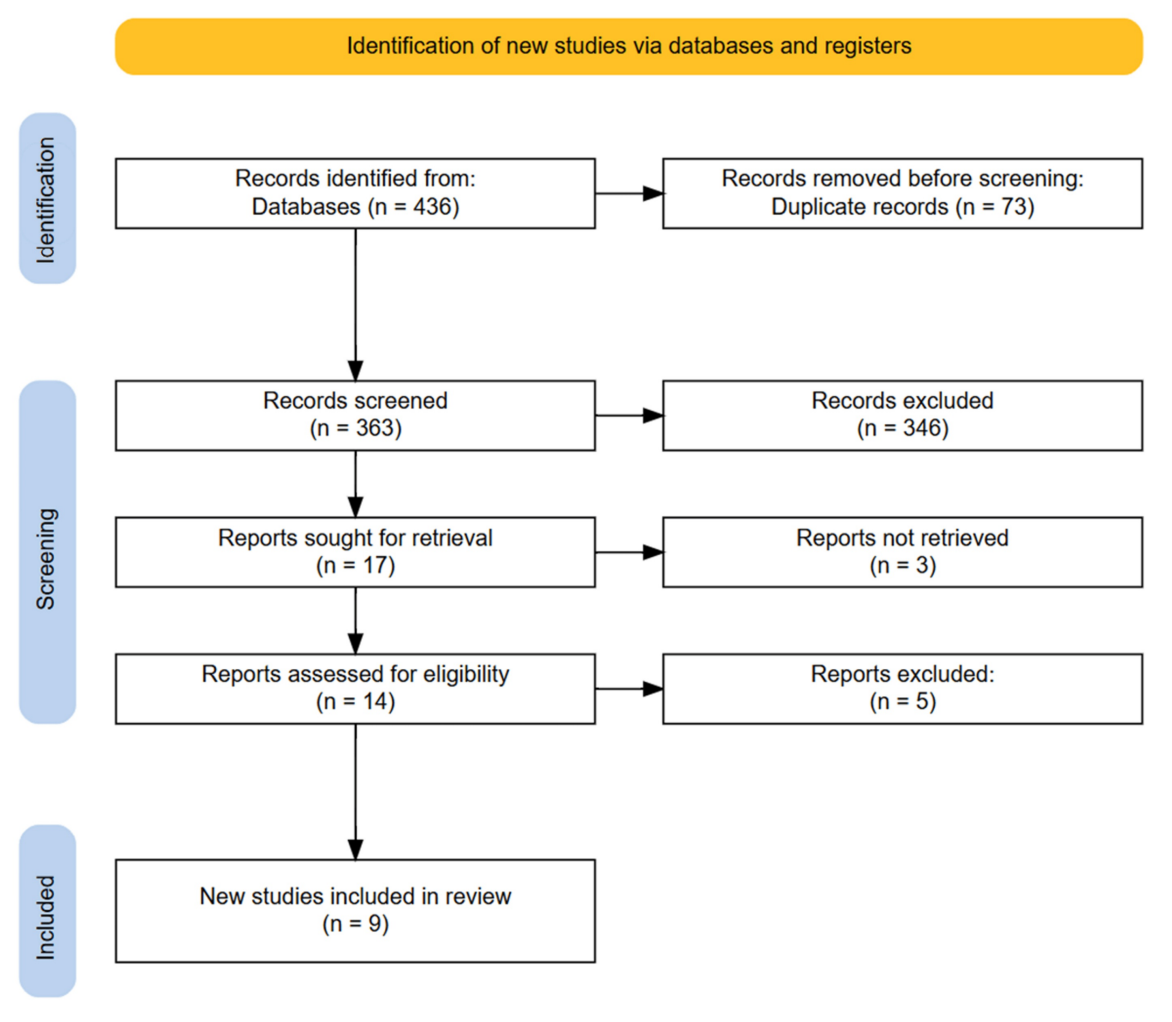
PRISMA diagram illustrating the study selection process. PRISMA: Preferred Reporting Items for Systematic reviews and Meta-Analyses

The database search initially identified 436 articles. After removing 73 duplicate entries, 363 studies underwent title and abstract screening. A total of 17 potentially eligible systematic reviews were assessed through full-text evaluation, ultimately resulting in the inclusion of nine high-quality reviews. No additional studies were identified through reference list screening. The PRISMA flowchart visually represents the study selection process (Figure 1).

Study Characteristics

This umbrella review included nine high-quality reviews published between 2020 and 2024 focusing on various DDMTs. These reviews synthesized evidence from 17-54 primary studies, predominantly randomized controlled trials (RCTs), with sample sizes ranging from small pilot studies (8-17 participants) to larger trials (up to 6,204 participants across all studies). Studies primarily included adults with T2DM, with mean ages ranging from 42.3 to 65 years, though some reviews also incorporated T1D populations. The reviewed digital interventions encompassed mHealth applications, CGM systems, telemedicine platforms, and AI-driven tools. Most reviews established specific inclusion criteria targeting home-based diabetes management interventions published within the last 5-10 years. Primary outcome measures consistently included HbA1c changes, with secondary outcomes including FBG, PPG, medication adherence, and psychosocial metrics. The methodological quality varied across primary studies, with some reviews noting limitations in study design, intervention duration (ranging from three to 12 months), and inconsistent reporting standards, highlighting the need for more rigorous and standardized approaches in future research (Table 1).

**Table 1:** A summary of the characteristics and main findings of included studies. T1DM: type 1 diabetes mellitus; T2DM: type 2 diabetes mellitus; HbA1c: glycated hemoglobin; BCTs: behavior change techniques; BG: blood glucose; BP: blood pressure; SBP: systolic blood pressure; DBP: diastolic blood pressure; LDL: low-density lipoprotein; HDL: high-density lipoprotein; CGM: continuous glucose monitoring; BMI: body mass index; MD: mean difference; CI: confidence interval; SMD: standardized mean difference; mHealth: mobile health; RCTs: randomized controlled trials

Authors	Year	Databases searched	Search duration	Number of studies included	Study design of included studies	Sample size	Population characteristics	Digital technologies studied	Main findings	Conclusions
El-Gayar et al. [9]	2021	PubMed/Medline and Web of Science	January 2010 to October 2020	21 studies (24 interventions)	RCTs	Total participants: 1,920 (1,040 in mHealth interventions and 880 in control groups)	Adults (over 18 years) diagnosed with T1DM or T2DM. Mean age: 51.2 years (range 32.9-68.1)	mHealth apps	mHealth apps led to statistically significant clinical outcomes compared to standard care for glycemic control (-0.38, 95% CI = -0.50 to -0.25, p < 0.0001), indicating a reduction in HbA1c. Using BCTs. "Action planning" and "Self-monitoring of outcome(s) of behavior" were the most effective BCTs. Interventions using behavior theory were not statistically different from those that did not (p = 0.18)	The meta-analysis provides evidence that mHealth is likely beneficial for diabetes patients when the right BCTs are applied. Further investigation into the role of theory in mHealth app-based intervention design is warranted
Kerr et al. [10]	2024	Embase, Medline, Central	Search conducted on April 5, 2022	28	Conducted on April 5, 2022. 23 RCTs (82%), 2 non-randomized comparative studies (7%), and 3 other designs (cross-sectional, prospective cohort, and retrospective cohort) (11%)	Average: 202 patients; median: 143 patients; range: 17-772	Mean age: 55.7 years; median age: 54.3 years; 51% female (range 29%-100%); ethnicities included Black, Chinese, Korean, and White; mean disease duration: 7.9 years (range 2.6-14 years); mean baseline HbA1c: 8.6% (range 6.8%-10.9%)	Digital glucose monitoring technologies (CGM or self-monitoring devices), connected scales, and accelerometers for physical activity monitoring combined with human coaching components	Digital interventions reduced HbA1c by 0.31% compared to usual care (95% CI -0.45% to -0.16%; p < 0.001). Higher-intensity interventions (with more personalized coaching) showed greater reductions (-0.45%) compared to medium-intensity (-0.29%) and low-intensity (-0.28%) interventions	Reducing HbA1c levels in individuals with T2DM using digital interventions is feasible, effective, and acceptable. The key feature of effective digital health interventions was the availability of timely and responsive personalized coaching by a dedicated healthcare professional
Lee and Kim [11]	2024	PubMed, CINAHL, DBpia, RISS	January 2016-August 2021	12	Various designs including RCTs	8-215 participants across studies	Primarily adults with T1DM and T2DM (11 studies on adults, 1 on adolescents); 16.7% T1DM, 50% T2DM, 33.3% both types	Mobile applications including Gamelet, DIABETEYAR, MyT1DHero, Medisafe, Switch, Intelligent Diabetes Management, BetaMe/Melon, SocialDiabetes app, Smart Glucose Manager, BlueStar mobile, My Care Hub	Mobile diabetes apps improved blood sugar levels and provided convenient user experience; most apps showed significant HbA1c reduction (0.3%-1.3% decrease in most studies); apps were rated highly for usability, ease of use, and satisfaction	Mobile apps are effective tools for patient-led self-management; usability should be evaluated using ISO9241-11 or MARS; HbA1c and self-management should be included as evaluation variables; more RCTs on app effectiveness are needed
Lee et al. [12]	2023	Medline, Embase, and PubMed	From inception till May 31, 2021	16	RCTs	6,204 (3,257 intervention; 2,947 control)	Older adults with T2DM with a mean age of 65 years or older	mHealth interventions including telemonitoring, telecommunication, online education programs, and wearable devices	Significant benefits HbA1c (MD = −0.24%; 95% CI: −0.44, −0.05; p = 0.01), postprandial BG (−2.91 mmol/L; 95% CI: −4.78, −1.03; p = 0.002), and triglycerides (−0.09 mmol/L; 95% CI: −0.17, −0.02; p = 0.010). No significant effects on LDL cholesterol, HDL cholesterol, BP, or BMI	Among older adults with T2DM, mHealth interventions were associated with improved cardiometabolic outcomes versus usual care. mHealth efficacy can be improved as current development is in its infancy. Addressing barriers such as technological frustrations may help strategize approaches to increase uptake and efficacy of mHealth interventions among older adults with T2DM
Liu et al. [13]	2020	Medline, Cochrane Library, Embase, and CINAHL Plus	January 2007 to January 2019	27	RCTs	Total participants across the 27 trials: median 75 per trial (range 14-250)	T2DM (19 trials), hypertension (6 trials), both T2DM and/or hypertension (1 trial), coexisting T2DM and hypertension (1 trial); mean age 57.3 years (range 48.4-69.5); median 54% male (range 28%-76%)	Mobile app-assisted self-care interventions with various features: monitoring (BG, BP, medication, body weight, diet, physical activity, mood), personalized feedback (automated feedback, medication adjustment aid, personalized goal setting, reminders), communication with healthcare providers, education materials, and data visualization	Mobile app interventions significantly reduced HbA1c (SMD -0.44, 95% CI: -0.59 to -0.29)	Mobile app-assisted self-care interventions can be effective tools for managing BG and BP. Their effectiveness likely stems from facilitating remote management of health issues and data, providing personalized self-care recommendations, enabling patient-provider communication, and supporting decision-making. More studies are needed to determine which combinations of features are most effective. Evidence regarding effects on behavioral, knowledge, and psychosocial outcomes remains scarce, warranting further examination
Natale et al. [14]	2023	Medline, Embase, PsycINFO, CINAHL	From inception to April 19, 2023	54 articles (56 citations)	Qualitative studies (33 used semi-structured/in-depth interviews, 14 used focus groups, 1 used both interviews and focus groups, 4 used open-ended questionnaires, 2 were document analyses)	1,845 participants (5 studies did not report number of participants)	Adults (18-91 years) with diabetes; 8 studies (15%) only included T2DM, 3 studies (6%) included both T1DM and T2DM, 3 studies (5%) did not report diabetes type, remaining studies included T1DM	CGM including flash CGM and real-time CGM and sensor-augmented insulin pump therapy	Six themes were identified: (1) gaining control and convenience, (2) motivating self-management, (3) providing reassurance and freedom, (4) developing confidence, (5) burdened with device complexities, and (6) excluded by barriers to access	CGM can improve self-management and confidence in patients managing diabetes. However, technical issues, uncertainty in readings, and cost may limit uptake. Education and training from health professionals may help reduce practical and psychological burden for better patient outcomes
Stevens et al. [15]	2020	PubMed, manual search of reference lists, Google Scholar	June 2010 to June 2020	25	RCTs	3,360 patients (1,735 in intervention groups, 1,626 in control groups)	Participants with T1DM (4 studies), T2DM (20 studies), and prediabetes (1 study); mean age: 52.1 years in the intervention group, 52.0 years in the control group; average diabetes duration: 12.49 years in the intervention group, 11.7 years in the control group	mHealth, mobile apps for self-management, DHTs, wearable sensors, web portals, smartphone applications	Overall improvement in HbA1c compared with usual care: MD of -0.56% for T1DM, -0.90% for T2DM, and -0.26% for prediabetes. Reduction in HbA1c was observed in 23 intervention groups and 21 control groups across all studies	Digital health technologies (DHTs) may reduce HbA1c levels in patients with T1DM, T2DM, and prediabetes. Further research is needed on clinical effectiveness beyond HbA1c, especially for T1DM and prediabetes, including measures of short-term glycemic variability and hypoglycemic events
Zhang et al. [16]	2022	PubMed, Web of Science, Cochrane Library, Embase, EBSCO, CNKI, Wanfang Data, VIP, and CBM	Database inception to August 2021	32	RCTs	Not specified for total (individual studies ranged across various sample sizes)	Adult patients (≥18 years) with T2DM in primary healthcare settings	Various telemedicine platforms including cell phones (17.5%), Internet (17.5%), text messaging (27.5%), apps (20%), glucose-monitoring devices (12.5%), and tablets (5%)	(1) Reduction in HbA1c, fasting glucose, and postprandial glucose after telemedicine intervention; (2) significant improvement in SBP and self-efficacy; (3) no significant improvement in weight, lipid metabolism, or diabetes awareness; (4) subgroup analysis showed significant improvement in HbA1c at 6 months of intervention	Telemedicine interventions may help patients with T2DM to effectively control BG and improve self-management in primary health care. Benefits are moderate and may not be sustained beyond 6 months. Evidence for improvement in lipid metabolism is insufficient, and further studies are needed
Zheng et al. [17]	2023	PubMed, CINAHL (EBSCO), Web of Science Core Collection, PsycINFO (Ovid), Embase (Ovid), and Scopus	January 2011 to June 2022	15	8 two-arm RCTs, 2 two-arm quasi-experimental studies, and 5 single-group interventions	Sample sizes ranged from 10 to 800	Adults with T2DM, aged 42.3-60.8 years, 25.9%-81.4% were females. In 3 studies reporting race, 15%-47.6% were non-White participants. 35.5%-100% took oral antihyperglycemic medication, 11.8%-37.8% used insulin, and 23.5%-30% had mixed oral medication and insulin	12 studies used smartphone apps with varied functions (meal photos, speech recognition, dietary assessment, nutrient intake displays, etc.), and 3 studies applied CGM	Mixed results: 9 of 12 pilot studies showed improved HbA1c; most resulted in varied dietary changes; few showed improved diabetes distress and depression. Only 3 studies were full RCTs with larger samples and 12-month duration	The application of mHealth technology for dietary intervention for adults with T2DM is still in the pilot testing stage. The preliminary effects are inconclusive on physiological, dietary behavioral, and psychosocial outcomes. Future full-scale studies are needed in more diverse populations

Quality assessment

Quality assessment of included articles using the AMSTAR 2 checklist showed that three of the included studies were of high quality, five were of moderate quality, and one was of low quality (Table 2).

**Table 2:** Quality assessment of included studies using the AMSTAR 2 checklist. AMSTAR: A MeaSurement Tool to Assess systematic Reviews

Authors	Overall quality assessment
El-Gayar et al. [9]	Moderate
Kerr et al. [10]	Moderate
Lee and Kim [11]	Low
Lee et al. [12]	High
Liu et al. [13]	High
Natale et al. [14]	Moderate
Stevens et al. [15]	Moderate
Zhang et al. [16]	High
Zheng et al. [17]	Moderate

Discussion

This systematic review synthesized evidence from nine high-quality reviews on the effectiveness of DDMTs for blood glucose control in patients with T2D at home. Our findings highlight that DDMTs, including mHealth applications, CGM systems, and telemedicine interventions, generally demonstrate positive effects on glycemic control, particularly in reducing HbA1c levels. Most studies reported statistically significant improvements in glycemic outcomes compared to standard care, with HbA1c reductions ranging from 0.24% to 0.90% [10,15]. These findings align with previous research suggesting that digital interventions can complement traditional diabetes management approaches by enhancing self-monitoring, facilitating timely feedback, and promoting patient engagement [18].

Several key factors appear to influence the effectiveness of DDMTs. First, the integration of personalized coaching and healthcare provider involvement was consistently associated with greater improvements in glycemic control. For instance, Kerr et al. found that higher-intensity interventions with more personalized coaching showed greater HbA1c reductions (-0.45%) compared to medium-intensity (-0.29%) and low-intensity (-0.28%) interventions [10]. This suggests that while technology provides the framework for self-management, human support remains crucial for optimizing outcomes. The synergistic relationship between digital tools and healthcare provider guidance underscores the importance of considering DDMTs as complementary to, rather than replacements for, traditional care models.

Behavior change techniques (BCTs) embedded within DDMTs also appear to play a significant role in their effectiveness. El-Gayar et al. identified "action planning" and "self-monitoring of outcome(s) of behavior" as particularly effective BCTs in mHealth interventions [9]. However, they noted that interventions using behavior theory were not statistically different from those that did not, highlighting the need for further investigation into how theoretical frameworks can best inform DDMT design. The incorporation of specific features such as real-time feedback, personalized goal setting, and reminders may enhance patient engagement and treatment adherence, ultimately contributing to improved glycemic outcomes.

The duration of interventions emerged as another important factor influencing DDMT effectiveness. Zhang et al. observed significant improvements in HbA1c at six months of intervention but noted that benefits may not be sustained beyond this period [16]. This temporal pattern suggests that while DDMTs can facilitate initial improvements in glycemic control, maintaining long-term engagement and adherence remains challenging. Future research should focus on strategies to sustain patient engagement with digital interventions over extended periods, possibly through adaptive designs that evolve with changing patient needs and preferences.

Patient perspectives and experiences with DDMTs, as explored by Natale et al., reveal both benefits and challenges [14]. Users reported gaining control and convenience, increased motivation for self-management, and greater confidence in managing their condition. However, they also experienced the burden of device complexities and barriers to access, including cost and technological literacy. These findings highlight the importance of user-centered design approaches that prioritize usability, affordability, and accessibility. Educational support and training from healthcare professionals may help reduce practical and psychological barriers to DDMT adoption and continued use.

Demographic considerations also warrant attention when assessing DDMT effectiveness. Lee et al. specifically examined mHealth interventions among older adults with T2DM and found significant benefits for HbA1c, postprandial blood glucose, and triglycerides [12]. This is noteworthy given that older adults are often underrepresented in digital health research and may face unique challenges related to technology adoption. However, addressing technological frustrations remains critical for increasing uptake and efficacy in this population. As digital health interventions continue to evolve, ensuring their accessibility and relevance across diverse age groups, socioeconomic backgrounds, and technological literacy levels will be essential for maximizing their impact on diabetes care.

Despite the generally positive findings, several limitations in the existing literature should be acknowledged. Many studies had relatively short follow-up periods, limiting our understanding of the long-term effectiveness of DDMTs. Sample sizes varied considerably across studies, with many involving relatively small cohorts, potentially limiting statistical power. Additionally, heterogeneity in intervention components, outcome measures, and reporting standards makes direct comparisons challenging. Zheng et al. noted that the application of mHealth technology for dietary intervention in T2DM is still primarily in the pilot testing stage, with preliminary effects being inconclusive across physiological, behavioral, and psychosocial outcomes [17].

Future directions and recommendations

From a clinical implementation perspective, several considerations emerge from our findings. Healthcare systems need to develop strategies for integrating DDMTs into routine care pathways, including appropriate patient selection, technology training, and ongoing support. Reimbursement models and cost-effectiveness analyses are needed to ensure sustainable implementation. Furthermore, data privacy and security concerns must be addressed to maintain patient trust in digital health solutions. Standardization of DDMT evaluation metrics would facilitate more robust comparisons across interventions and guide evidence-based selection of appropriate technologies for different patient populations [19].

Future research directions should include larger, more diverse study populations with longer follow-up periods to assess the sustainability of benefits. Studies should systematically evaluate which specific DDMT features and implementation approaches are most effective for different patient subgroups, considering factors such as age, technological literacy, socioeconomic status, and disease severity. More research is needed on the impact of DDMTs on psychosocial outcomes, quality of life, and healthcare utilization patterns. Additionally, exploring the potential of emerging technologies such as artificial intelligence and machine learning to enable more personalized and adaptive interventions represents a promising frontier in digital diabetes management.

## Conclusions

This umbrella systematic review provides substantial evidence supporting the effectiveness of DDMTs for improving glycemic control in the home-based management of T2D. The integration of digital tools with personalized coaching, appropriate BCTs, and consideration of patient experiences appears to optimize outcomes. However, challenges related to long-term engagement, technological accessibility, and implementation within healthcare systems remain. As technology continues to evolve, so too must our approaches to evaluating, implementing, and refining digital interventions for diabetes management. By addressing these challenges and building on the strengths identified in this review, DDMTs have the potential to significantly enhance diabetes self-management, improve clinical outcomes, and reduce the burden on healthcare systems.
